# Optimization of Ultrasonic Welding Process Parameters to Enhance Weld Strength of 3C Power Cases Using a Design of Experiments Approach

**DOI:** 10.3390/polym14122388

**Published:** 2022-06-13

**Authors:** Chil-Chyuan Kuo, Qing-Zhou Tsai, Ding-Yang Li, Yong-Xhi Lin, Wen-Xiong Chen

**Affiliations:** 1 Department of Mechanical Engineering, Ming Chi University of Technology, New Taipei City 24301, Taiwan; m10118022@mail2.mcut.edu.tw (Q.-Z.T.); m10118002@mail2.mcut.edu.tw (D.-Y.L.); 2 Research Center for Intelligent Medical Devices, Ming Chi University of Technology, New Taipei City 24301, Taiwan; 3 AcBel Polytech Inc., No. 84, Gungjuan Road, New Taipei City 243, Taiwan; dream@gmail.com (Y.-X.L.); jackchen@gmail.com (W.-X.C.)

**Keywords:** ultrasonic welding, plastics, Taguchi methods, power case, confirmation experiments

## Abstract

Ultrasonic welding (UW) is a joining of plastics through the use of heat generated from high-frequency mechanical motion, which is known as an efficient process in many applications, such as textile, packaging, or automotive. UW of thermoplastics has been widely employed in industry since no polymer degradations are found after UW. However, the trial-and-error approach is frequently used to study optimum UW process parameters for new 3C plastic power cases in current industry, resulting in random efforts, wasted time, or energy consumption. In this study, Taguchi methods are used to study optimum UW process parameters for obtaining high weld strength of a plastic power case. The most important control factor influencing the weld strength is amplitude, followed by weld pressure, hold time, and trigger position. The optimum UW process parameters are amplitude of 43.4 µm, weld pressure of 115 kPa, hold time of 0.4 s, and trigger position of 69.95 mm. Finally, the confirmation experiments are performed to verify the optimum process parameters obtained in this study.

## 1. Introduction

Ultrasonic welding (UW) [1] is an industrial process. UW is an efficient method of fusing molded thermoplastic [2] or metallic parts [3] using the energy from low-amplitude and high-frequency acoustic vibrations. UW offers three distinct advantages over other forms of welding: (a) UW produces a high-quality bond and a tight and clean seal, (b) UW saves production costs, and (c) UW saves time. The entire process can take just seconds. Zhi et al. [4] investigated the relations among the loss modulus of carbon-fiber-reinforced polyamide 66 composite and time for obtaining stable weld areas. It was found that peak load, weld area, and endurance limit of the double-pulse ultrasonic welding process weld joint increased by about 15%, 23%, and 59%, respectively. Additionally, the double-pulse ultrasonic welding process decreases variance in the strength of the joints. Fan et al. [5] achieved the microstructure homogenization of aluminum (Al) alloy weld seams by adding ultrasonic irradiation in metal inert gas welding. Results showed that ultrasonic cavitation is the main reason for the microstructure homogenization of columnar grains near the fusion line and conventional metal inert gas is completely transformed into the equiaxed grains under the action of ultrasonic irradiation. Additionally, the size of equiaxed grains is more refined than that of the initial columnar grains. Ni et al. [6] reviewed the current state of ultrasonic spot welding of Al to copper with numerous crucial issues containing plastic deformation, relative motion, vertical displacement, materials flow, and electrical conductivity. Das et al. [7] investigated the effects of process parameters on joint strength and process robustness when multi-layered joints of dissimilar metals are produced by ultrasonic metal welding. In addition, response surfaces are developed to identify the relationship and sensitivity between the output quality indicators and input process parameters. Alinaghian et al. [8] introduced a hybrid method called bending mode ultrasonic-assisted friction stir welding and investigated the effects under various vibration amplitudes on longitudinal residual stress in a cross-section area of 3 mm and thickness of 5 mm of Al plates using the contour method. Results suggested that ultrasonic-assisted friction stir welding with amplitude of about 3 µm gives the best outcome for the welding of thick joints. Yang et al. [9] analyzed the contact behavior and temperature characterization during welding using the harmonic balance method. It was found that there is a certain separation which is mainly friction heat generation in the early stage of welding. Dobrota et al. [10] optimized the parameters for the ultrasonic welding of two materials, namely 70% polybutylene terephthalate with 30% fiber glass and expanded polytetrafluoroethylene. The topography of the material layer from the plate-type part is analyzed. Qiu et al. [11] reviewed the advances of applying ultrasonic thermal welding by the third phase for thermoplastic materials to provide guidance for using ultrasonic thermal welding by the third phase in polymers. Jongbloed et al. [12] studied heating in the continuous ultrasonic welding of thermoplastic composites. Results showed that the higher temperatures at the welding interface in continuous ultrasonic welding are attributed to pre-heating of the energy director due to vibrations being transmitted downstream of the sonotrode. Micus et al. [13] focused on the formation of reliable connections between conductive textiles and conventional litz wires using ultrasonic welding. It was found that the resistance of joints increased more than 300% because silver-coated wires suffered under laundry cycles. Additionally, mechanical strength during the peeling test decreased by only about 20% after 15 cycles and remained the same after 30 cycles. Frederick et al. [14] characterized nanocomposite films containing multi-walled carbon nanotubes for thermo-electrical behavior to assess self-heating. The maximum temperature increased with multi-walled carbon nanotubes and film thickness. Staab et al. [15] investigated the potential and limitations of the technology as a non-destructive testing method. In addition, quantitative information on volume-specific proportions could be obtained and compared in relation to each other using a tool for 3D segmentation of the composition of the joining zone.

Taguchi methods were developed by Genichi Taguchi to improve the quality of manufactured goods, and more recently have also been applied to engineering, marketing, advertising, and biotechnology [16,17]. Mahmoudian et al. [18] performed polymerization of methyl methacrylate to improve interaction of the nanoparticles using the Taguchi methods. It was found that modified alumina nanoparticles had a better dispersion and interaction in comparison to unmodified alumina nanoparticles because of the modification procedure. Azadeh et al. [19] employed the Taguchi methods to select the optimum maintenance policy. Costa et al. [20] employed the Taguchi methods to optimize the process parameters for steel turning processes. Effertz et al. [21] employed the Taguchi methods to study process parameters for friction spot welded Al alloy. Akıncıoglu et al. [16] employed the Taguchi methods to investigate the effects of cryogenically treated tools in the turning of super alloy on surface roughness. Results showed that surface roughness of the super alloy can be improved greatly. Adnan et al. [17] used Taguchi methods to investigate the springback behavior of Al alloy strips with non-uniform thickness. Results showed that thickness is the most significant parameter to formability.

According to practical experience, drawbacks of the trial-and-error approach [22] include wasted time and random efforts. In this study, a cost-effective method for enhancing the weld strength of molded thermoplastic parts is proposed using the Taguchi methods [23]. Finally, the optimum process parameter was also verified by confirmation experiments.

## 2. Experimental Details

**Figure 1:**
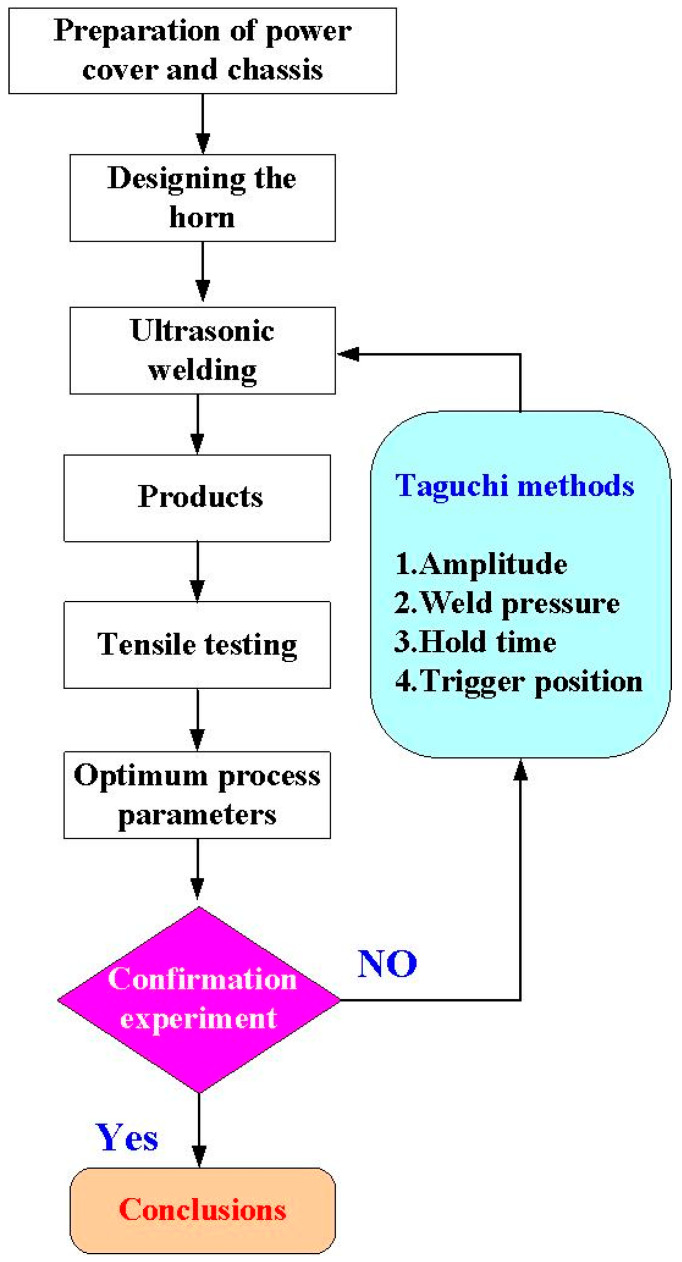
Flow diagram of the experimental methodology.

**Figure 2:**
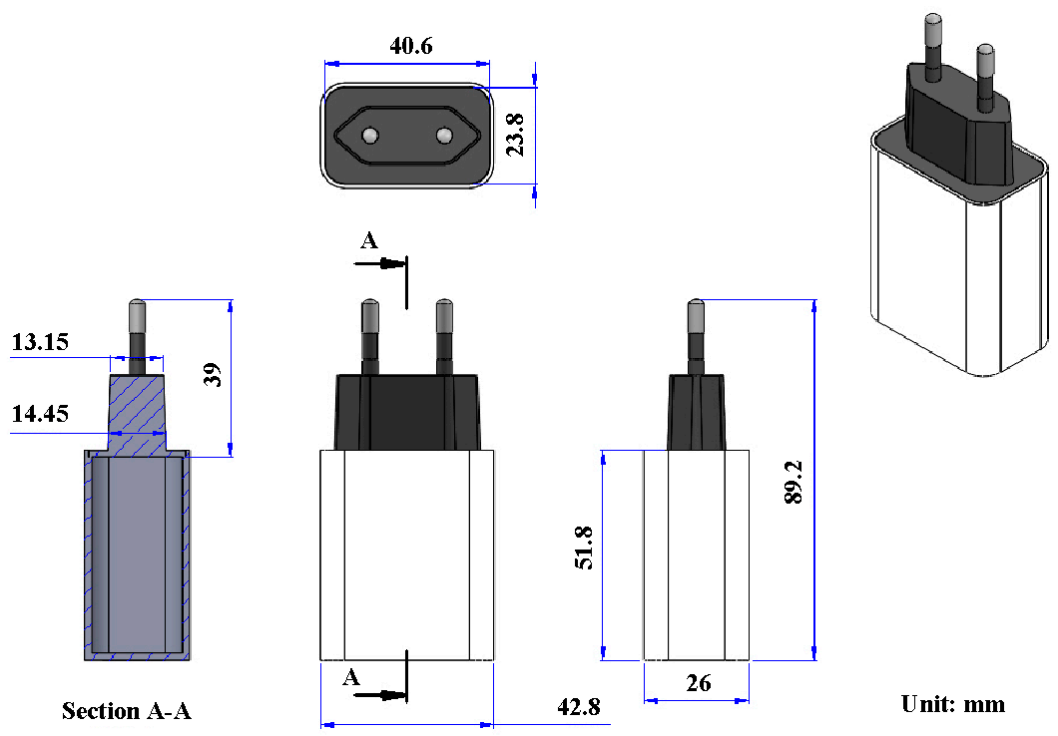
3D CAD model and dimensions of a power case.

Figure 1 shows the flow diagram of the experimental methodology. Figure 2 shows the 3D CAD model and dimensions of a power case.

**Figure 3:**
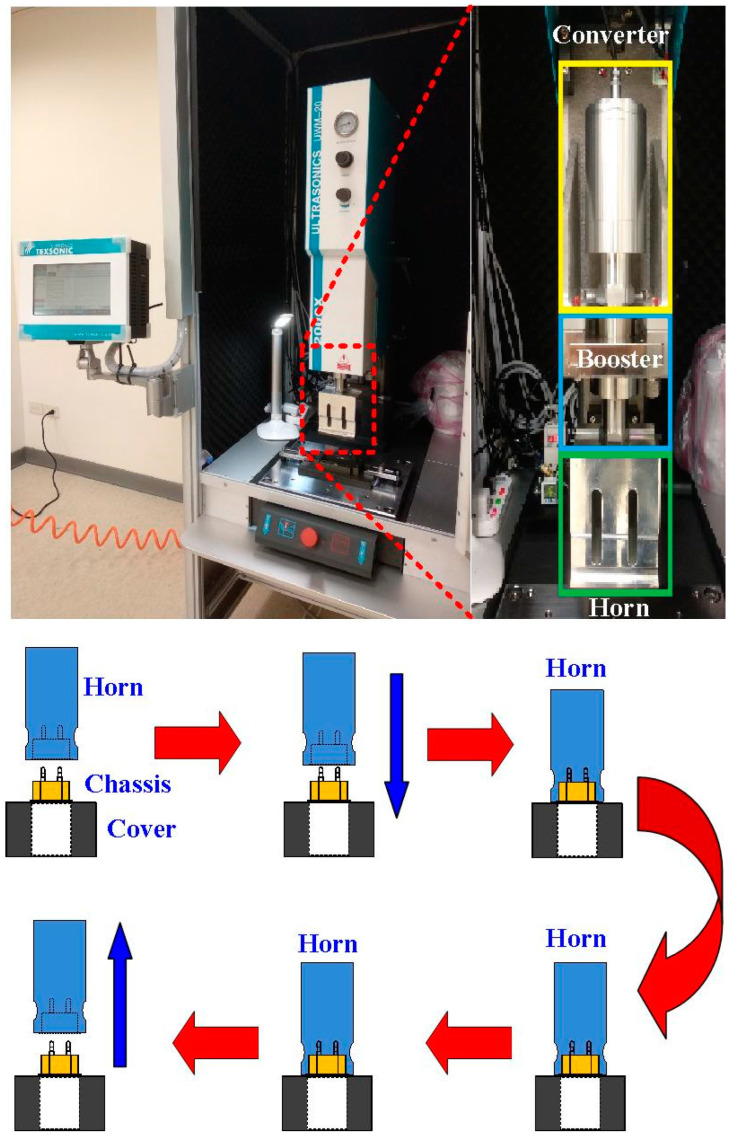
Photo of an ultrasonic plastic welding machine used in this study and schematic illustration of UW processes.

**Figure 4:**
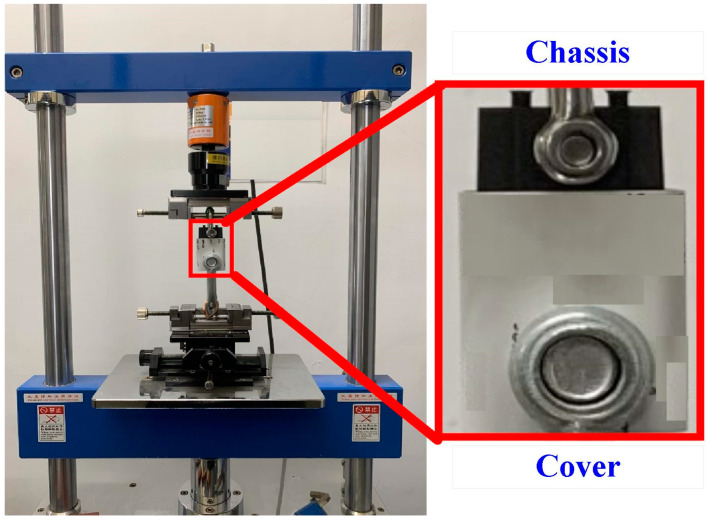
Photographic illustration of tensile testing conditions.

Both the cover and chassis of a 3C power case were fabricated by plastic injection molding via polycarbonate (PC) [24] using a plastic injection molding machine (KT2S, Kinki Inc., Taipei city, Taiwan) [25]. The process parameters involve injection pressure of 18 MPa, injection speed of 80 mm/s, and cooling time of 10 s. The length, width, and height of the cover are 42.8 mm, 26 mm, and 51.8 mm, respectively. The length, width, and height of the chassis are 40.6 mm, 23.8 mm, and 39 mm, respectively. The thickness of both the cover and chassis is about 2 mm. Figure 3 shows the photo of an ultrasonic plastic welding machine (UWM 2000X, Texsonic Inc., Furth, Germany) used in this study and schematic illustration of UW processes. After UW, the weld strength of the power case was investigated using a tensile test apparatus (1220WS. Se testststems Inc., Taipei city, Taiwan). Figure 4 shows the situation of the tensile testing. Tensile test speed of 50 mm/min was selected for the investigation of strain rate. The strain rate is about 900 S^−1^ based on the stress to strain curve.

To study the effects of process parameters of UW on the weld strength of the injection molded PC parts, the Taguchi methods [26] with Latin square 9 orthogonal design were used to determine the signal-to-noise (S/N) ratio in this study. It is well known that the Taguchi methods have three different kinds of quality characteristics, i.e., the-nominal-the-best, the-larger-the-better, and the-smaller-the-better [27]. Equations (1)–(3) represent the-larger-the-better, the-smaller-the-better, and the-nominal-the-best, respectively. Three different S/N quality characteristic formulations are shown in the following equations. To investigate the optimum process parameters for fabricating a power case with the highest weld strength, the orthogonal array (OA) [28] was employed in this study since it is suitable for the four process control factors with three levels. In general, analysis of variance (ANOVA) is frequently used to compare the difference between the means of the groups. The ANOVA table involves various statistics, including sum of square, degree of freedom, *p*-value, as well as contribution ratio of each control factor. Finally, the confirmation experiment is performed to validate the optimum process parameters of UW obtained in this study.

(1) $$\mathrm{The}\text{-}\mathrm{smaller}\text{-}\mathrm{the}\text{-}\mathrm{better}\;S/N=-10\;\log\; [\frac{1}{n}\sum_{i=1}^{n}\left(y_{i}{}^{2}\right)]$$

(2) $$\mathrm{The}\text{-}\mathrm{larger}\text{-}\mathrm{the}\text{-}\mathrm{better}\;S/N=-10\;\log\; [\frac{1}{n}\sum_{i=1}^{n}\left(\frac{1}{y_{i}{}^{2}}\right)]$$

(3) $$\mathrm{The}\text{-}\mathrm{nominal}\text{-}\mathrm{the}\text{-}\mathrm{best}\;S/N=-10\;\log\; [\frac{\bar{y}}{s^{2}y}]$$

where *s*^2^*y* is the variance, $\bar{y}$ is the average, *n* is the number of observations, and *y* is the observed data. 

## 3. Results and Discussion

### 3.1. Amplitude

**Figure 5:**
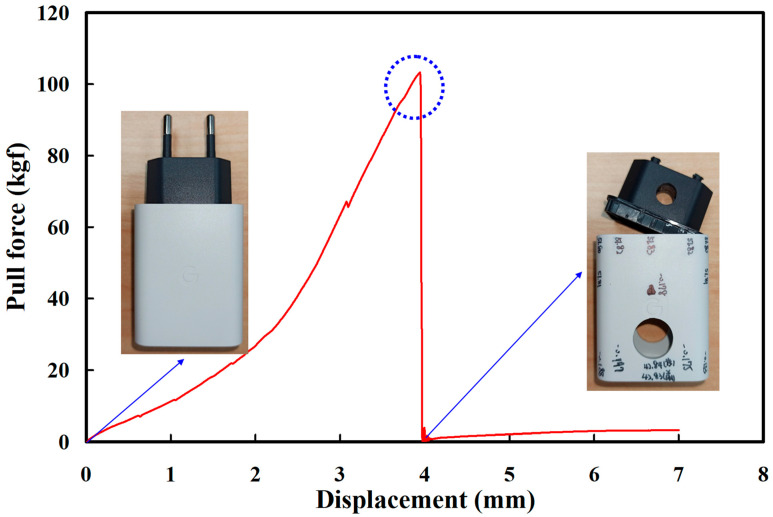
Result of the tensile testing.

**Figure 6:**
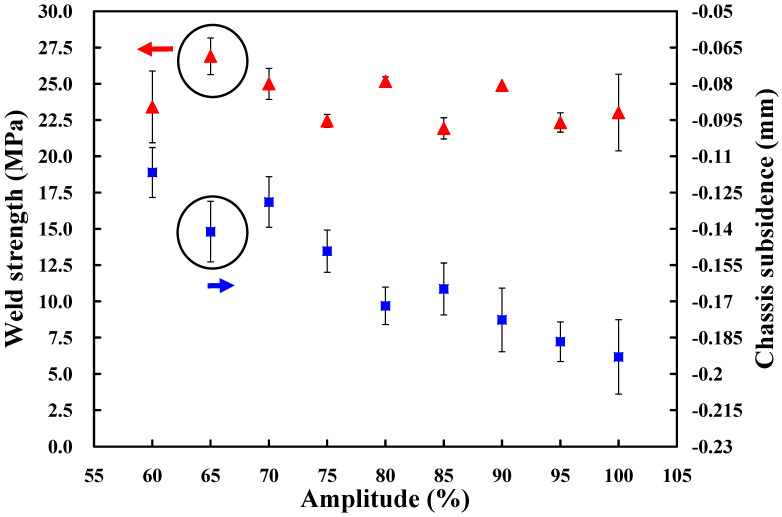
Effects of the different amplitudes on weld strength and chassis subsidence.

In this study, four process parameters influencing the UW quality of a power case are selected as control factors based on the fishbone diagram. The four control factors include amplitude [29], weld pressure [30], trigger position [31], and hold time [32]. Firstly, the one-factor-at-a-time method [33] is used to investigate third levels of the four control factors. It should be noted that the-larger-the-better is used to determine the best combination of parameters for UW of power covers since higher tensile force stands for better weld strength of the power cover after UW. According to the conventional trial-and-error method, the weld strength of a power case is related to the trigger position, hold time, amplitude, and weld pressure. Firstly, the trigger position of 70 mm, hold time of 0.5 s, and weld pressure of 100 kPa are fixed to study the optimum amplitude. To investigate the effect of amplitude on the weld strength of a power case, nine different kinds of amplitudes are performed in this study. Specifically, amplitude of 100% in the UW machine is 62 µm because the amplitude of the oscillator used in this study is 20 µm. The magnifications of the amplifier and welding head are 2 and 1.55, respectively. Thus, the amplitudes of 60%, 65%, 70%, 75%, 80%, 85%, 90%, 95%, and 100% in the UW machine stand for the amplitude values of 37.2 µm, 40.3 µm, 43.4 µm, 46.5 µm, 49.6 µm, 52.7 µm, 55.8 µm, 58.9 µm, and 62 µm, respectively. To reduce experimental error, each UW process parameter was tested with five test specimens. Figure 5 shows the result of the tensile testing. Note that the weld strength of a 3C power case can be estimated form the results of the tensile testing. Figure 6 shows the effects of the different amplitudes on weld strength and chassis subsidence. In general, higher weld strength and lower chassis subsidence represent better welding quality for a 3C power case using UW. Based on the cause-and-effect analysis, it was found that an amplitude of 65% seems to be the optimum parameter. Thus, the amplitude of 65%, i.e., amplitude of 40.3 µm, is determined as level 2 of control factor 1. The amplitudes of 60% and 70%, i.e., amplitudes of 37.2 µm and 43.4 µm, are determined as levels 1 and 3 of control factor 1, respectively.

### 3.2. Weld Pressure

**Figure 7:**
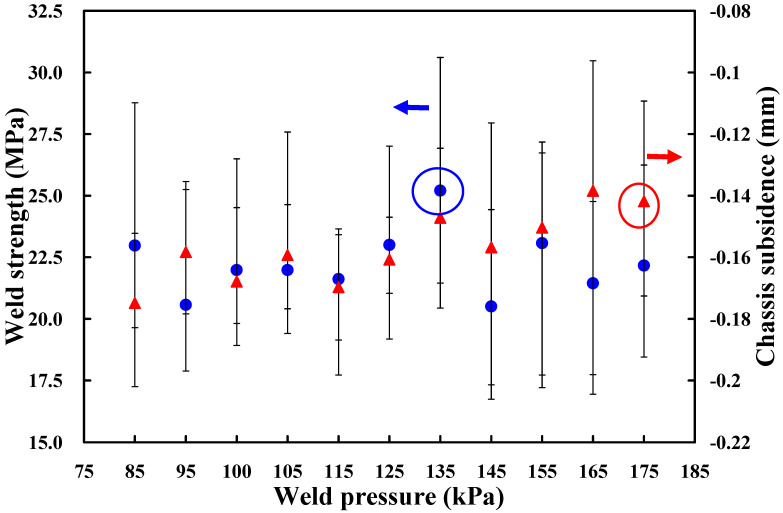
Effects of different weld pressures on weld strength and chassis subsidence.

Based on the above results, the amplitude of 65%, i.e., amplitude of 40.3 µm, trigger position of 70 mm, and hold time of 0.5 s are fixed. To investigate the effect of weld pressure on the weld strength of a power case, eleven different weld pressures, i.e., 85 kPa, 95 kPa, 100 kPa, 105 kPa, 115 kPa, 125 kPa, 135 kPa, 145 kPa, 155 kPa, 165 kPa, and 175 kPa are performed in the following experiments. Figure 7 shows the effects of the different weld pressures on weld strength and chassis subsidence. It was found that the weld pressure of 125 kPa seems to be the optimum parameter based on higher weld strength and lower chassis subsidence. Thus, the weld pressure of 125 kPa is determined as level 2 of control factor 2. The weld pressures of 115 kPa and 135 kPa are determined as levels 1 and 3 of control factor 2, respectively.

### 3.3. Trigger Position

**Figure 8:**
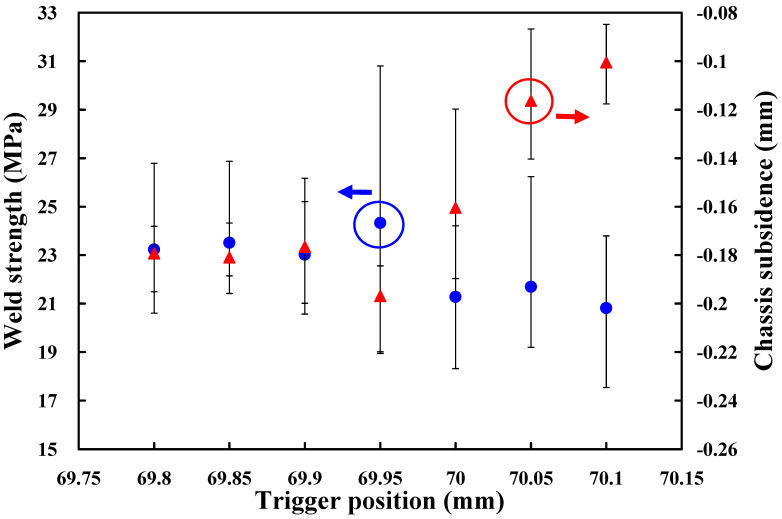
Effects of the different trigger positions on weld strength and chassis subsidence.

Based on the above results, the amplitude of 65%, i.e., amplitude of 40.3 µm, weld pressure of 125 kPa, and hold time of 0.5 s are fixed. To investigate the effect of trigger position on the weld strength of a power case, seven different trigger positions, i.e., 69.8 mm, 69.85 mm, 69.9 mm, 69.95 mm, 70 mm, 70.05 mm, and 70.1 mm are performed in the following experiments. Figure 8 shows the effects of the different trigger positions on weld strength and chassis subsidence. According to both weld strength and chassis subsidence, it was found that the trigger position of 69.95 mm seems to be the optimum parameter. Thus, the trigger position of 69.95 mm is determined as level 2 of control factor 3. The trigger positions of 69.9 mm and 70 mm are determined as levels 1 and 3 of control factor 3, respectively.

### 3.4. Hold Time

**Figure 9:**
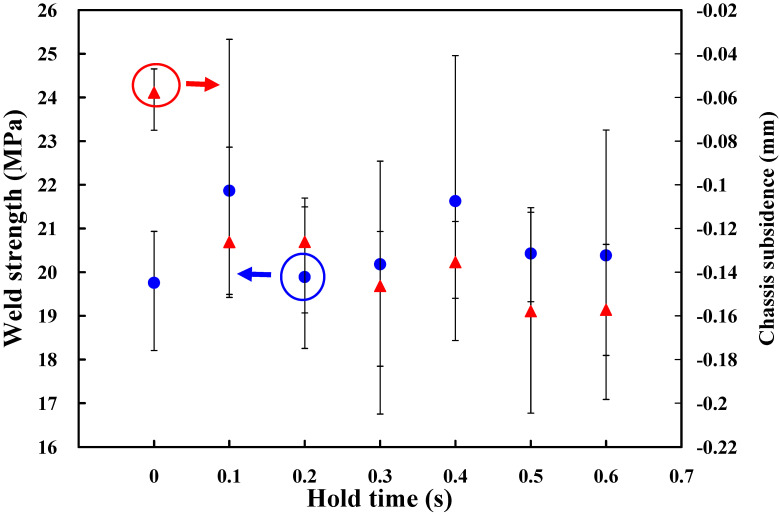
Effects of the different hold times on weld strength and chassis subsidence.

Based on the above results, the amplitude of 65%, i.e., amplitude of 40.3 µm, weld pressure of 125 kPa, and trigger position of 69.95 mm are fixed. To investigate the effect of hold time on the weld strength of a power case, seven different hold times, i.e., 0 s, 0.1 s, 0.2 s, 0.3 s, 0.4 s, 0.5 s, and 0.6 s are performed in the following experiments. Figure 9 shows the effects of the different hold times on weld strength and chassis subsidence. According to both weld strength and chassis subsidence, it was found that the hold time of 0.4 s seems to be the optimum parameter. Thus, the hold time of 0.4 s is determined as level 2 of control factor 4. The hold times of 0.3 s and 0.5 s are determined as levels 1 and 3 of control factor 4, respectively. According to the experimental results described above, four process control factors and their levels are summarized in Table 1. 

**Figure 10:**
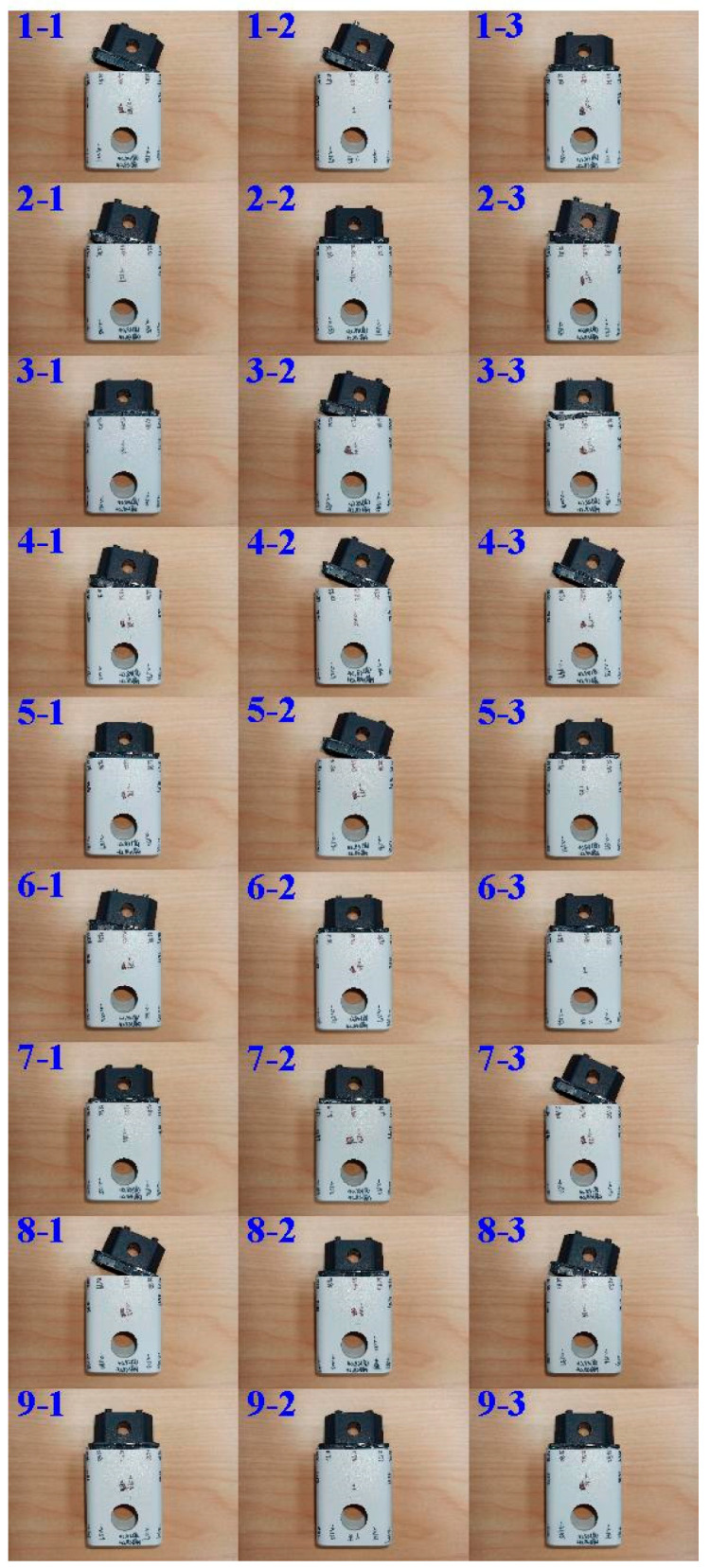
Results of tensile testing for parts fabricated by different process parameters.

**Figure 11:**
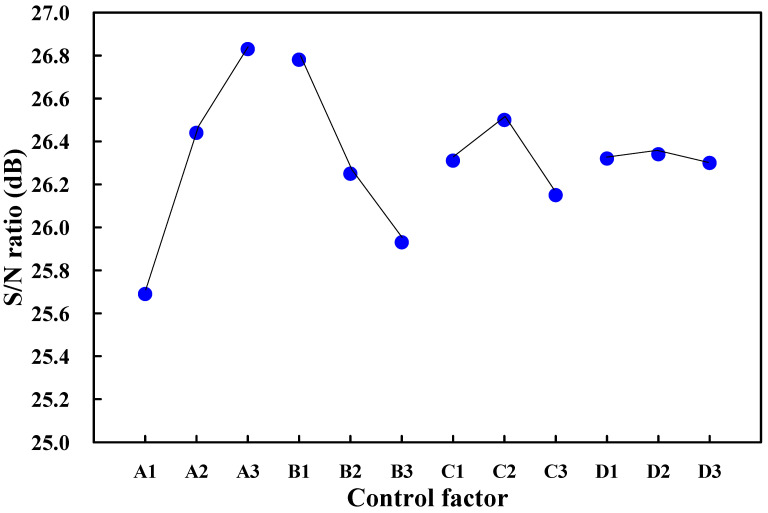
S/N ratio effects of each process control factor.

Table 2 shows the tensile testing results. In this study, the-larger-the-better is used since higher weld strength means better welding quality of UW. Figure 10 shows the results of tensile testing for the parts fabricated by different process parameters. Table 3 shows the response table of S/N ratio based on the-larger-the-better quality characteristics. Figure 11 shows the S/N ratio effects of each process control factor. As can be seen, an optimum combination of process control factors and levels can be determined based on the higher S/N ratio. The best combination of control factor levels for fabricating a power case with high weld strength is A3, B1, C2, and D2, i.e., amplitude of 70%, weld pressure of 115 kPa, hold time of 0.4 s, and trigger position of 69.95 mm.

**Figure 12:**
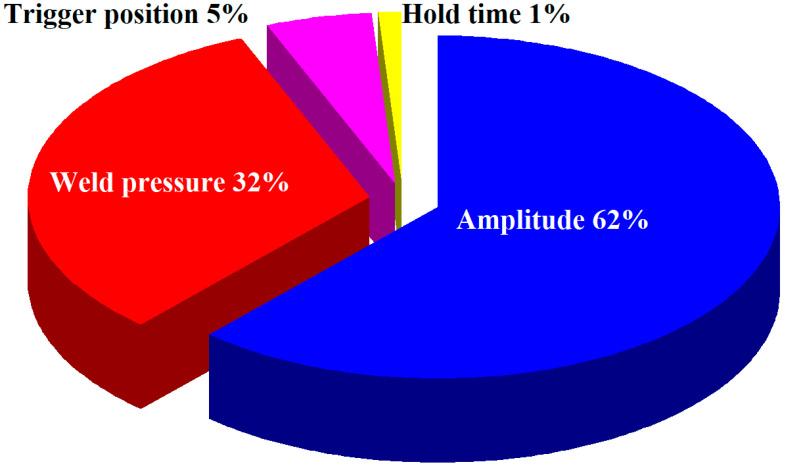
Schematic illustration of the percentage of contribution.

The results of ANOVA are summarized in Table 4. Note that the most important control factor influencing weld strength is the amplitude, which has a contribution of approximately 62%. The contributions of weld pressure, hold time, and trigger position are about 32%, 5%, and 1%, respectively. Figure 12 shows the schematic illustration of the percentage of contribution. Thus, the optimum UW process parameters of a new 3C power case involve amplitude of 43.4 µm, weld pressure of 115 kPa, hold time of 0.4 s, and trigger position of 69.95 mm. 

**Figure 13:**
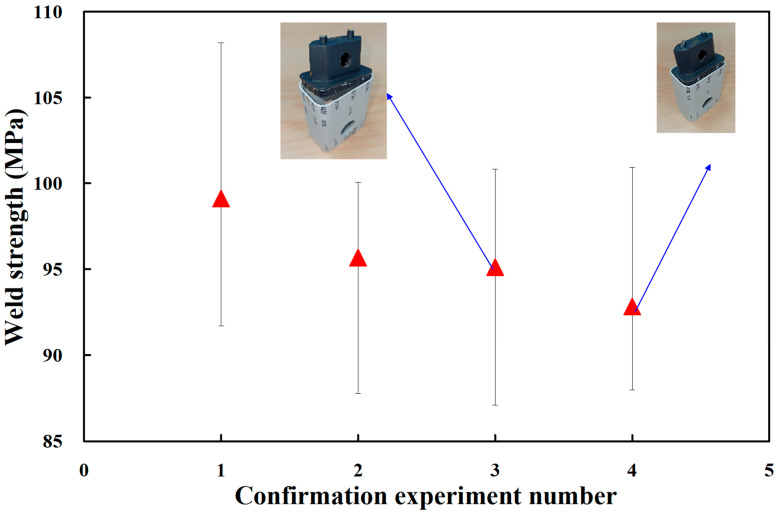
Results of confirmation experiments.

To verify the optimum UW process parameters obtained in this study, three sets of non-optimal process parameters for UW were employed randomly in the confirmation experiment. Figure 13 shows the results of the confirmation experiments. Table 5 shows the results of verifying the optimum process parameters. As can be seen, the weld strengths of the four plastic power cases are approximately 18.85 MPa, 18.19 MPa, 18.09 MPa, and 17.66 MPa, respectively. Results revealed that the average weld strength of a plastic power case obtained by optimum UW process parameters is significantly higher than the average weld strength of a plastic power case obtained by general process parameters of UW. Based on the results described above, the remarkable findings of this study are very practical and provide the greatest application potential in the 3C industry. In this study, the material of the power case used was PC. A wide range of thermoplastic materials [34], including acrylonitrile butadiene styrene (ABS) [35] or PC/ABS [36] can also be used to joint a 3C power case. Laser welding [37,38] can also be employed for jointing 3C power cases because it is a high speed, precise, and clean manufacturing process. Unfortunately, this study did not investigate the tensile fracture mechanisms of welded parts fabricated with different UW process parameters. Therefore, scanning electron microscopy can be used to study the tensile fracture mechanism and surface morphology of the tensile fracture surface. These issues are currently being investigated and the results will be presented in a later study.

## 4. Conclusions

UW is accomplished by converting high-frequency electrical energy into high-frequency mechanical motion. The mechanical motion creates frictional heat for forming the molecular bond between the plastic parts. According to practical experience, UW is a promising approach for welds between dissimilar materials. The main conclusions from the experimental work in this study are as follows:

1. The remarkable findings in this study are very practical and provide potential applications in industry because investigation of optimum UW process parameters for a new 3C plastic power case in current industry is possible.
2. The most important control factor influencing weld strength is amplitude, followed by weld pressure, hold time, and trigger position.
3. Confirmation experiments were performed to verify the obtained optimum process parameters. The optimum UW process parameters are amplitude of 43.4 µm, weld pressure of 115 kPa, hold time of 0.4 s, and trigger position of 69.95 mm.

## Figures and Tables

**Table 1:** Process control factors and their levels.

Control Factor		Level 1	Level 2	Level 3
A	Amplitude (%)	60	65	70
B	Weld pressure (kPa)	115	125	135
C	Hold time (s)	69.9	69.95	70
D	Trigger position (mm)	0.3	0.4	0.5

**Table 2:** Tensile testing results.

Experiment No.	Control Factor				Weld Strength (MPa)			σ^2^	S/N (dB)
	A	B	C	D	1	2	3		
1	A1	B1	C1	D1	18	22	21.6	2.23	26.14
2	A1	B2	C2	D2	20.2	22.4	17.1	2.65	25.82
3	A1	B3	C3	D3	22.8	21.6	13.9	4.82	25.11
4	A2	B1	C2	D3	24.3	23.9	20.2	2.27	27.06
5	A2	B2	C3	D1	24.6	18.1	20.1	3.36	26.21
6	A 2	B3	C1	D2	20.9	19.5	19.9	0.72	26.06
7	A3	B1	C3	D2	24.3	20.8	23.6	1.90	27.14
8	A3	B2	C1	D3	22.7	18.9	25	3.09	26.74
9	A3	B3	C2	D1	19.5	20.8	24.9	2.79	26.61

**Table 3:** Response table of S/N ratio based on the-larger-the-better quality characteristics.

Control Factor	Level 1	Level 2	Level 3
Amplitude (%)	25.69	26.44	26.83
Weld pressure (kPa)	26.78	26.25	25.93
Hold time (s)	26.31	26.50	26.15
Trigger position (mm)	26.32	26.34	26.30

**Table 4:** ANOVA table.

Control Factor		Leve1	Level 2	Level 3	Sum of Squares	Degree of Freedom	Mean Squares	Contribution (%)
A	Amplitude (%)	25.69	26.44	26.83	2.018	2	1.009	62
B	Weld pressure (kPa)	26.78	26.25	25.93	1.106	2	0.553	32
C	Hold time (s)	26.31	26.50	26.15	0.177	2	0.089	5
D	Trigger position (mm)	26.32	26.34	26.30	0.002	2	0.001	1

**Table 5:** Results of verifying optimum process parameters.

Confirmation Experiment Number	UW Process Parameters	Weld Strength (MPa)					
		1	2	3	4	5	Average
1 (Optimum process parameters)	Amplitude 43.4 µm	18.80	17.44	17.81	20.56	19.61	18.85
	Weld pressure 115 kPa						
	Trigger position 69.95 mm						
	Hold time 0.4 s						
2	Amplitude 37.2 µm	16.69	18.526	18.51	18.20	19.03	18.19
	Weld pressure 135 kPa						
	Trigger position 69.95 mm						
	Hold time 0.3 s						
3	Amplitude 40.3 µm	16.56	18.57	19.17	18.46	17.66	18.09
	Weld pressure 125 kPa						
	Trigger position 69.90 mm						
	Hold time 0.5 s						
4	Amplitude 43.4 µm	17.33	16.73	17.19	19.19	17.81	17.66
	Weld pressure 125 kPa						
	Trigger position 70.00 mm						
	Hold time 0.3 s						

## Data Availability

Data and materials are available.
